# Editorial: UN world AIDS day, a neuroscience perspective

**DOI:** 10.3389/fncel.2023.1288615

**Published:** 2023-10-03

**Authors:** Carmen C. Diaconu, Ronald J. Ellis, Dirk M. Hermann

**Affiliations:** ^1^Stefan S. Nicolau Institute of Virology, Romanian Academy, Bucharest, Romania; ^2^Departments of Neurosciences and Psychiatry, University of California, San Diego, San Diego, CA, United States; ^3^Department of Neurology, University Hospital Essen, University of Duisburg-Essen, Essen, Germany

**Keywords:** acquired immunodeficiency syndrome, HIV/AIDS, human immunodeficiency virus, inflammation, neuro-cognitive, neural complications associated with HIV, opportunistic infections

Initiated in 1988, World AIDS Day inaugurated the first global health awareness day, creating an opportunity to forge a unified international coalition in the fight against human immunodeficiency virus (HIV). Since then, December 1st has become a day of solidarity with the ~39 million individuals currently living with HIV and a time of remembrance for those who have passed due to AIDS-related illnesses, which marks the collaborative engagement on various HIV-related themes by United Nations entities, governments, and civil society. HIV, primarily known for its impact on the immune system, can have widespread effects on the central nervous system (CNS) and peripheral nervous system (PNS), and can be responsible for mood disorders, cognitive and motor deficits as a consequence of inflammation that can damage neurons and supporting cells, increase the susceptibility to opportunistic infections, and increase the risk of vascular diseases, including cerebrovascular diseases and stroke.

Even with virally suppressive highly active antiretroviral therapy (HAART, often used as combination antiretroviral therapy, cART) as the standard treatment for HIV, neurological issues remain a problem to understand and solve. HAART effectively reduces the HIV load within the body and mitigates the advancement of HIV-associated neurological diseases. On the other hand, long-term use of HAART, and particularly cART, can also have neurological side effects. Nevertheless, further research is imperative to delineate the underlying mechanisms that could be targeted, aiming at developing more potent and better tolerable treatments. Having a neuroscience perspective on HIV infection, this Research Topic represents a platform to assemble latest research on HIV's effect on the nervous system and neural complications associated with HIV.

Drug consumption is a common comorbidity among people with HIV that is associated with exacerbated CNS conditions. Both HIV and addictive substances can individually weaken the immune system. When combined, they can have a synergistic effect, further compromising immune function. This can lead to difficulties in controlling HIV replication, increased susceptibility to opportunistic infections, neuroinflammation, HIV-associated neurocognitive disorders (HAND), and potentially accelerated progression to AIDS. In this Research Topic, Basova et al. focused on the inflammatory and signaling pathways that are triggered within innate immune cells due to the interplay between HIV peptides (particularly HIV-1 Tat) and the effect of methamphetamine (Meth). The authors demonstrated that Meth, which is known to accentuate HIV-associated neurological disorders, increased the transcription of inflammatory genes in interaction with HIV-1 Tat peptide via inducing reactive oxygen species (ROS). HIV-1 Tat did not further impact the direct effects of ROS in the context of Meth but was able to promote gene activity independently from ROS via additional transcription factors. HIV-1 Tat decreased the expression of anti-oxidant genes and may contribute to the exacerbation of oxidative stress induced by ROS in response to Meth. Understanding the modulation of inflammatory mechanisms by HIV peptides and exposure to addictive substances, such as Meth, could help enhancing therapeutic options for individuals infected with HIV, regardless of their substance abuse history.

As a consequence of opportunistic infections, HIV-induced immunosuppression can lead to poor neurological outcomes. Ene et al. described a cluster of HIV-infected children and adolescents with severe immunosuppression exhibiting a novel syndrome of myoclonic epilepsy that occurred during measles outbreaks in Romania. After rapid progression this form of subacute myoclonic measles encephalitis was typically fatal. However, three patients with higher CD4 counts at onset and slower progression of neurological symptoms seemed to benefit from immune deficiency treatment with cART. Prompt initiation of antiretroviral treatment, which restores an effective immune response, might be key to subacute myoclonic measles encephalitis survival.

In their study, Brkic et al. explored the role of cART in controlling the process of progressive neurodegeneration with primary focus on uncovering potential correlations of cART efficacy parameters in CNS with neurometabolic profiles on multi-voxel magnetic resonance spectroscopy (MRS) as a non-invasive tool for neuroinflammation assessment. The authors could not show significant correlations between indices of cART efficacy in CNS and neurometabolic profiles obtained using multi-voxel MRS. However, their findings indicated a noteworthy correlation between monocyte efficacy (ME) scores and neuronal markers exclusively within the dorsal anterior and posterior cingulate gyrus and the parietal subcortical white matter. This observation indirectly supports the notion that the impact of cART on stopping the progressive neurodegeneration primarily affects metabolically active brain regions that play a role in cognitive functioning. Identifying non-invasive tools and markers for neuroinflammation assessment and monitoring the effectiveness of antiretroviral drugs in the CNS remain priorities, especially with the increasing life expectancy of persons with HIV.

A prolonged lifespan also increases the risk of age-related neurodegenerative diseases such as Alzheimer's disease (AD) and its precursor, amnestic mild cognitive impairment (aMCI). Fields et al. evaluated the relationship between brain complement protein levels, beta-amyloid plaques, and neurocognitive impairment in persons with HIV. Their findings align with earlier results showing an association between complement system proteins and the neuropathogenesis of both HAND and AD. This could potentially indicate the complement system as a promising target for therapeutic interventions.

Major depressive disorder (MDD) and HAND are the prevailing neuropsychiatric challenges encountered by persons with HIV. Both MDD and HAND have significant morbidity and premature mortality, and they exhibit some common neurobehavioral features, notably apathy. Goodkin et al. reviewed the overlapping features of MDD and HAND, analyzing possible shared pathophysiological mechanisms and advancing the hypothesis that neuroinflammation and dopamine depletion contribute to the development of both disorders. Further research will be needed to explore the underlying causes and dedicated treatments for subjects suffering from MDD and HAND. Castaneda et al. examined if the brain's reactions to rewards could offer insights into the nature of apathy in persons with HIV. The authors found that the P300 potential measured by EEG holds promise as a biomarker for motivated behavior in older subjects with HIV.

Important insights of this Research Topic relate to mechanisms underlying neuroinflammation, metabolic dysfunction, cognitive and mood disturbances. Despite undoubted achievements in therapeutic possibilities brought about by HAART and cART, World AIDS Day reminds us not to forget about persistent challenges in HIV/AIDS management.

## Author contributions

CCD: Writing—original draft, Writing—review and editing. RJE: Writing—review and editing. DMH: Writing—review and editing.

